# WMC-RTDETR: a lightweight tea disease detection model

**DOI:** 10.3389/fpls.2025.1574920

**Published:** 2025-05-06

**Authors:** Youcheng Zhang, Jun Song, Xinjian Yu, Xingfu Ji

**Affiliations:** ^1^ College of Information Science and Technology, Nanjing Forestry University, Nanjing, China; ^2^ Electronic Information Department, Jiangsu JITRI Intelligent Sensing Technology Co., Ltd., Nantong, Jiangsu, China

**Keywords:** tea pest and disease detection, RT-DETR, wavelet transform, multiscale multihead self-attention, contextual feature reconstruction, embedded deployment

## Abstract

Tea pest and disease detection is crucial in tea plantation management, however, challenges such as multi-target occlusion and complex background impact detection accuracy and efficiency. To address these issues, this paper proposes an improved lightweight model, WMC-RTDETR, based on the RT-DETR model. The model significantly enhances the ability to capture multi-scale features by introducing wavelet transform convolution, improving the feature extraction accuracy in complex backgrounds, and increasing detection efficiency while reducing the number of model parameters. Combined with multiscale multihead self-attention, global feature fusion across scales is realized, which effectively overcomes the shortcomings of traditional attention mechanisms in small target detection. Additionally, a context-guided spatial feature reconstruction feature pyramid network is designed to refine the target feature reconstruction through contextual information, thereby improving the robustness and accuracy of target detection in complex scenes. Experimental results show that the proposed model achieves 97.7% and 83.1% respectively in mAP50 and mAP50:95 indicators, which outperform the original model. In addition, the number of parameters and floating-point operations are reduced by 35.48% and 40.42% respectively, enabling highly efficient and accurate detection of pests and diseases in complex scenarios. Furthermore, this paper successfully deploys the lightweight model on the Raspberry Pi platform, which proves that it has good real-time performance in resource-constrained embedded environments, providing a practical solution for low-cost disease monitoring in agricultural scenarios.

## 1 Introduction

Tea is a traditional Chinese cash crop with high market demand (Zhuo et al., 2024). However, tea cultivation faces significant threats by pests and diseases such as tea anthracnose. Currently, traditional methods for detecting pests and diseases relies on manual labor, which is inefficient and subject to variability due to operator expertise and environmental conditions. Therefore, it is of great significance to develop techniques that can realize timely and accurate detection of tea pests and diseases. Such advancements can help mitigate production losses and enhance the economic outcomes for tea farmers (Hua, 2023).

With the rapid development of computer vision technology, many researchers have combined image processing with machine learning for automated identification of tea pests and diseases. For example, Xie et al. constructed a full-wavelength based Extreme Learning Machine (ELM) classifier model to recognize early and late blight on tomato leaves by extracting texture features at five effective wavelengths (Xie et al., 2015). Hu et al. proposed a feature segmentation and enhancement method that combined support vector machine (SVM) and generative adversarial network (C-DCGAN), and then used the VGG16 model to identify tea diseases (Hu et al., 2019a). Hossain et al. developed an image processing system based on Support Vector Machines (SVM), which classifies the uploaded images of tea diseases by comparing them with the features in the database (Hossain et al., 2018). Although traditional machine learning methods have shown significant advantages over manual inspection in identifying tea plant diseases and insect pests, they are constrained by limitations, such as the complex manual feature extraction process, high model complexity, and slow processing speed.

In contrast, deep learning has gained attention due to its high prediction accuracy and end-to-end autonomous learning capability. Convolutional Neural Networks (CNNs), a typical structure of deep learning, have proven to be highly effective for feature extraction compared to shallow machine learning approaches. The development of image recognition technology has further promoted the widespread application of CNN in automatic image classification and plant diseases detection (Kattenborn et al., 2021). For example, Hu et al. added a multi-scale feature extraction module and depth-separable convolution to the traditional CNN model to identify lesion points on leaves more efficiently (Hu et al., 2019b). Li et al. proposed a Faster regional convolutional neural network (Faster R-CNN) applied in agricultural greenhouse environments, which showed significant results in detecting a variety of micro-pests (Li et al., 2021). Su et al. used the feature pyramid network (FPN) in the ResNet-101 network as the backbone network of the masked region convolutional neural network (Mask R-CNN) and successfully achieved accurate recognition of wheat Fusarium wilt (FHB) (Su et al., 2020). In these studies, scholars have used CNN to automatically extract the characteristics of crop diseases. The accuracy of CNN-based image recognition methods has been significantly improved compared to traditional machine learning methods. However, such models are usually highly complex and difficult to meet real-time requirements.

In recent years, with the introduction of YOLO (You Only Look Once) series (Redmon et al., 2016), the one-stage detection network model has demonstrated higher efficiency than the traditional two-stage detection model and has achieved remarkable results in the field of image recognition. Wu et al. proposed a cost-effective drone target detection method based on YOLOv3 for the early diagnosis of pine wilt disease (PWD) (Wu et al., 2021). Xue et al. integrated the self-attention mechanism and various improvement modules based on YOLOv5, which significantly improved the accuracy and efficiency of tea pest and disease detection (Xue et al., 2023). Wang et al. constructed a new multi-scale feature fusion module based on the improved YOLOv6 to enhance the feature fusion expression, thereby improving the detection performance and generalization ability of diseased tomato leaves (Wang et al., 2024e). Ye et al. developed a small-target disease detection method based on YOLOv8, which successfully solved the challenges of complex tea disease backgrounds, difficulties in detecting small lesions and low recognition rates of similar phenotypic symptoms (Ye et al., 2024).

Although the single-stage detection architecture of the YOLO model enables it to complete target localization and classification in a single forward propagation with high real-time detection capability, there are still some limitations when dealing with targets in unstructured environments, which are susceptible to factors such as light changes, foliage occlusion and complex backgrounds. In scenarios involving mutual target occlusion or complex backgrounds, non-maximal suppression (NMS) is often required to address overlapping detection frames. This not only increases the inference time, but also requires manual tuning of hyper-parameters to balance speed and accuracy.

To address these issues, a novel end-to-end model DETR (Detection Transformer) was proposed (Carion et al., 2020). The original intention of DETR was to simplify the target detection process and eliminate the dependence on anchor points and NMS, but its inference speed and real-time detection capabilities have not yet fully met actual needs. Subsequently, the Real Time-Detection Transformer (RT-DETR) model was optimized based on the DETR architecture by introducing a newly designed hybrid encoder to efficiently handle multi-scale features (Zhao et al., 2024). Compared with YOLOv8, RT-DETR shows superior detection accuracy and stability in complex environments, especially in multi-target detection and tasks with complex relationships between targets. Feng et al. proposed a new framework for fusion of multi-source forest remote sensing data that combines soft thresholding and cascaded group attention (CGA) modules based on RT-DETR to further improve the accuracy and robustness of the target recognition task (Feng et al., 2024). Wang et al. proposed a lightweight algorithm called PDSI-RTDETR for tomato maturity detection, which combines high accuracy and fast response with model lightweighting, showing potential in detecting different maturity levels of tomatoes (Wang et al., 2024d). Wang et al. proposed a lightweight UAV aerial infrared small target detection algorithm that can effectively capture small target features and ensure recognition accuracy even in complex environments and long target distances (Wang et al., 2024c). While RT-DETR has been successfully applied in diverse object detection tasks, its use in tea disease identification remains underexplored, highlighting an area of opportunity for further research.

To address the challenges in tea disease detection, this paper proposes a lightweight tea disease detection model based on the improved RT-DETR. The contributions of this paper are as follows:

(1) Wavelet transform convolution (WTConv) for backbone enhancement: An enhanced WTConv_Block module is constructed by integrating wavelet transform convolution with the residual block. This improvement not only reduces the number of model parameters, but also enhances the model’s ability to capture multi-scale features and improves overall robustness.
(2) Introduction of multiscale multihead self-attention (M2SA) module: The multiscale multihead self-attention module is integrated into the attention-based intrascale feature interaction module (AIFI), replacing the traditional multi-head attention mechanism to form the M2SA-AIFI module. This method realizes cross-scale global feature fusion, which effectively address the limitations of the traditional attention mechanism in small target detection and multi-scale scenarios, and thus locates the key regions in the image more accurately.
(3) Lightweight neck frame design: By introducing the independently designed context-guided spatial feature reconstruction feature pyramid network (CSRFPN) into the cross-scale feature-fusion module (CCFF), the spatial features of the target are reconstructed using context information. This design significantly improves the detection capability of multi-scale targets while maintaining detection accuracy, and effectively reduces the model’s parameter count.
(4) Deployment on Raspberry Pi: The improved lightweight model is deployed on the low-cost embedded platform Raspberry Pi, achieving efficient operation in an environment with limited hardware resources while ensuring high detection accuracy. This shows that the improved model is suitable for both high-performance hardware platforms and resource-limited embedded systems, providing a practical and cost-effective disease detection and monitoring systems in the agricultural field.

## 2 Methods and materials

### 2.1 Material preparation

#### 2.1.1 Dataset collection

**Figure 1:**
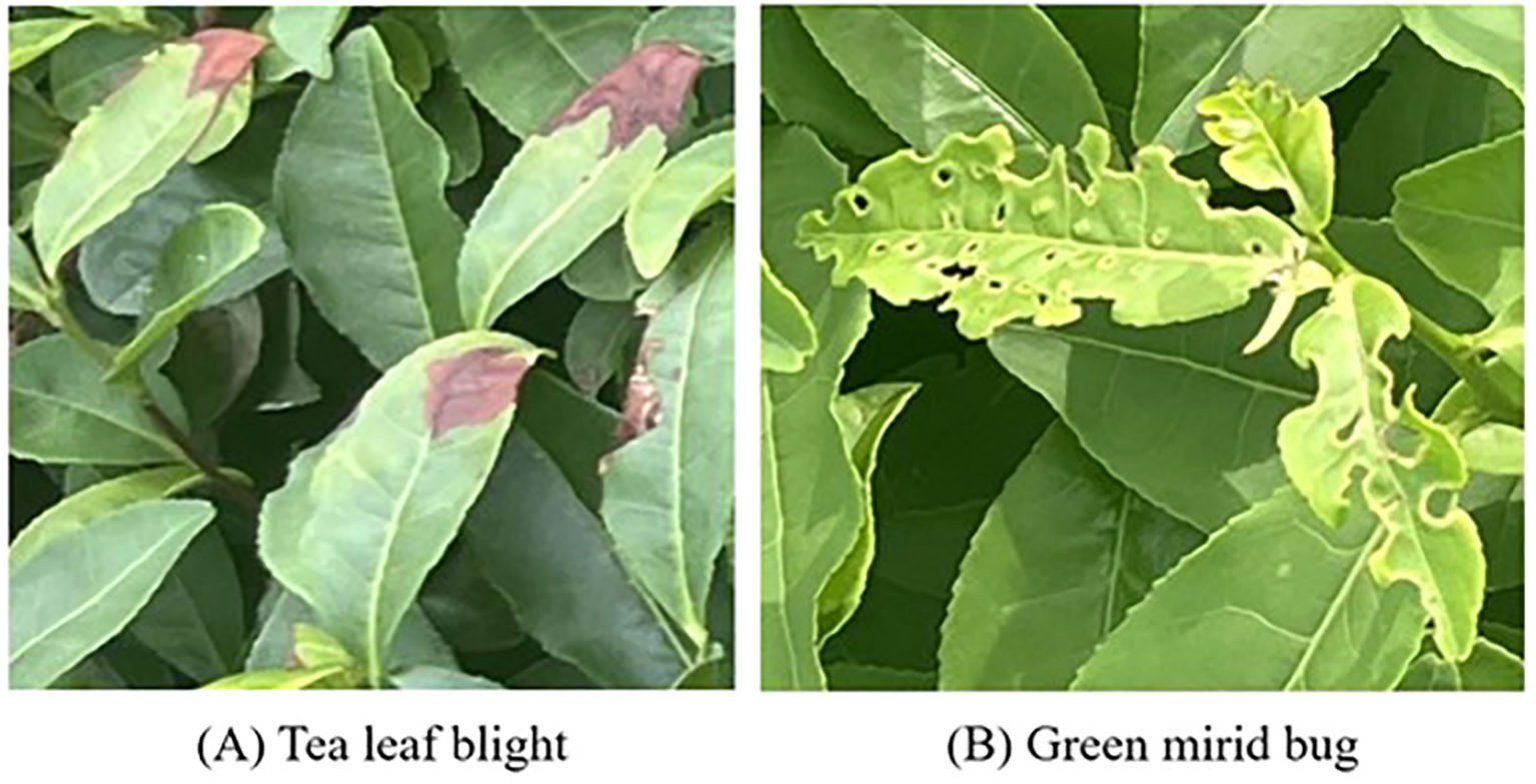
Some typical sample images. The bug **(A)** Tea leaf blight, **(B)** Green mirid.

This study collected images of tea tree pests and diseases in a natural environment. The data collection took place at Maoshan Tea Garden, Jurong City, Zhenjiang City, Jiangsu Province, China, July 6, 2024. We captured images with a resolution of 3060*4080 using our phone Honor 50 in full sunlight. All data was collected manually. The captured images cover scenarios with dense and sparse disease areas, and leaf occlusion and overlap are obvious in some photos. The main types of pests and diseases include tea leaf blight and green mirid bug. **Figure 1** shows some typical sample images in our dataset.

#### 2.1.2 Dataset division

**Figure 2:**
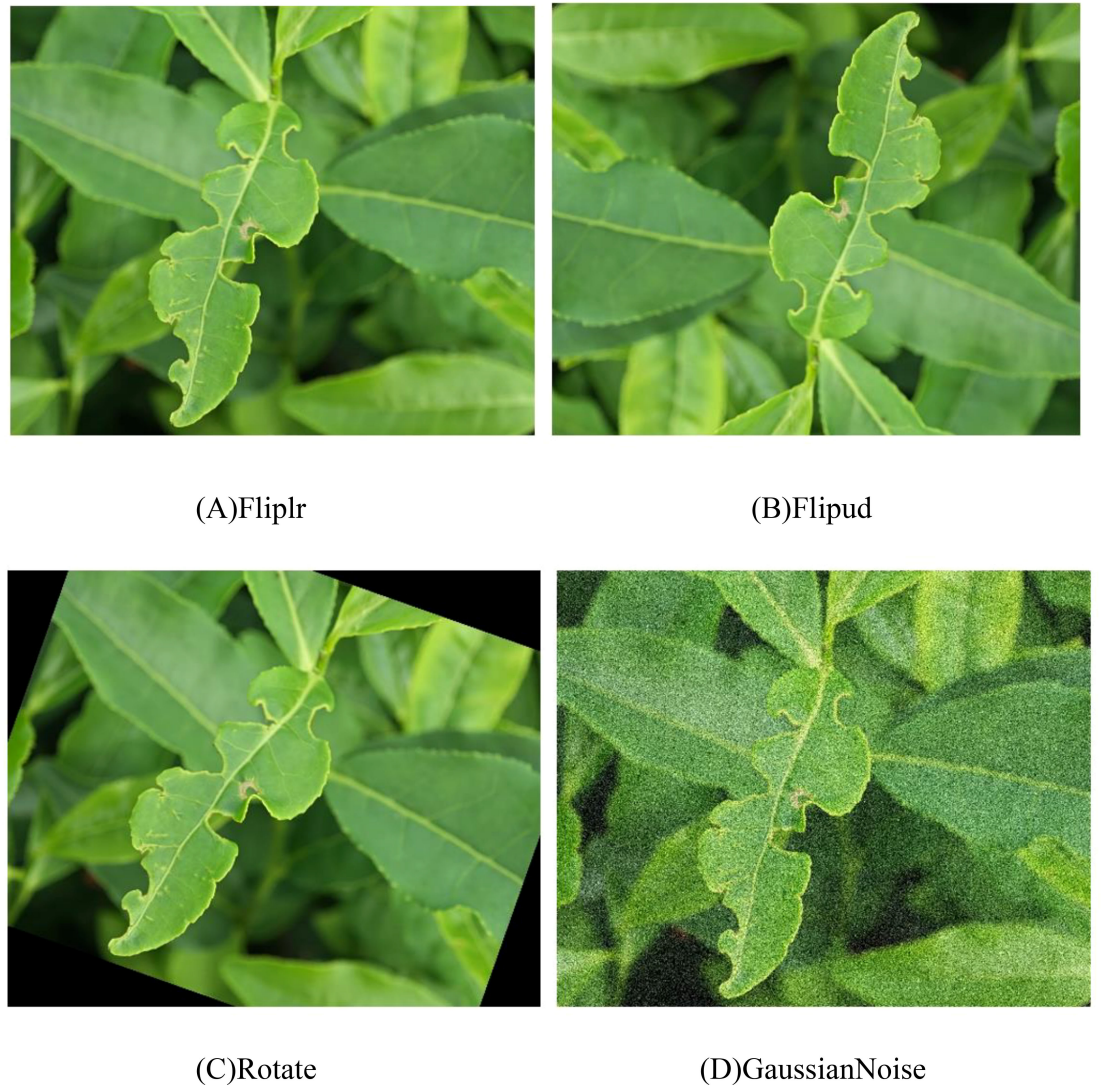
Data enhancement. **(A)** Fliplr, **(B)** Flipud, **(C)** Rotate, **(D)** Gaussian Noise.

First, we manually screened all the photos we took and selected a total of 160 images containing tea leaf blight and green mirid bugs. Next, we used the Labelimg to annotate all images for subsequent model training and evaluation. To improve the accuracy of the experiments and ensure the quality of the dataset, we performed data augmentation on these annotated 160 images and expanded them to 1280 images. Data enhancement methods include horizontal flipping, vertical flipping, rotation, and addition of Gaussian noise, as shown in **Figure 2**. The enhanced dataset is divided into training set, validation set and test set in a ratio of 7:1:2, specifically the training set contains 896 images, the validation set contains 256 images, and the test set contains 128 images. After data augmentation, it contains a total of 1808 tags of tea leaf blight and 2952 tags of green mirid bugs.

### 2.2 Algorithm design

#### 2.2.1 RT-DETR

RT-DETR is a novel real-time end-to-end target detection module. It introduces the Efficient Hybrid Encoder, which combines attention-based intrascale feature interaction module (AIFI) with Convolution-based cross-scale feature-fusion module (CCFF). This design effectively reduces computational overhead and improving the efficiency of multi-scale feature processing, enabling RT-DETR to achieve the goal of real-time detection while maintaining high detection accuracy. Additionally, RT-DETR reduces the training time without the use of mosaic data enhancement strategies and significantly improves the detection accuracy while maintaining similar detection speed. The architecture of RT-DETR mainly consists of three core parts: backbone network, efficient hybrid encoder and decoder with auxiliary prediction head.

**Figure 3:**
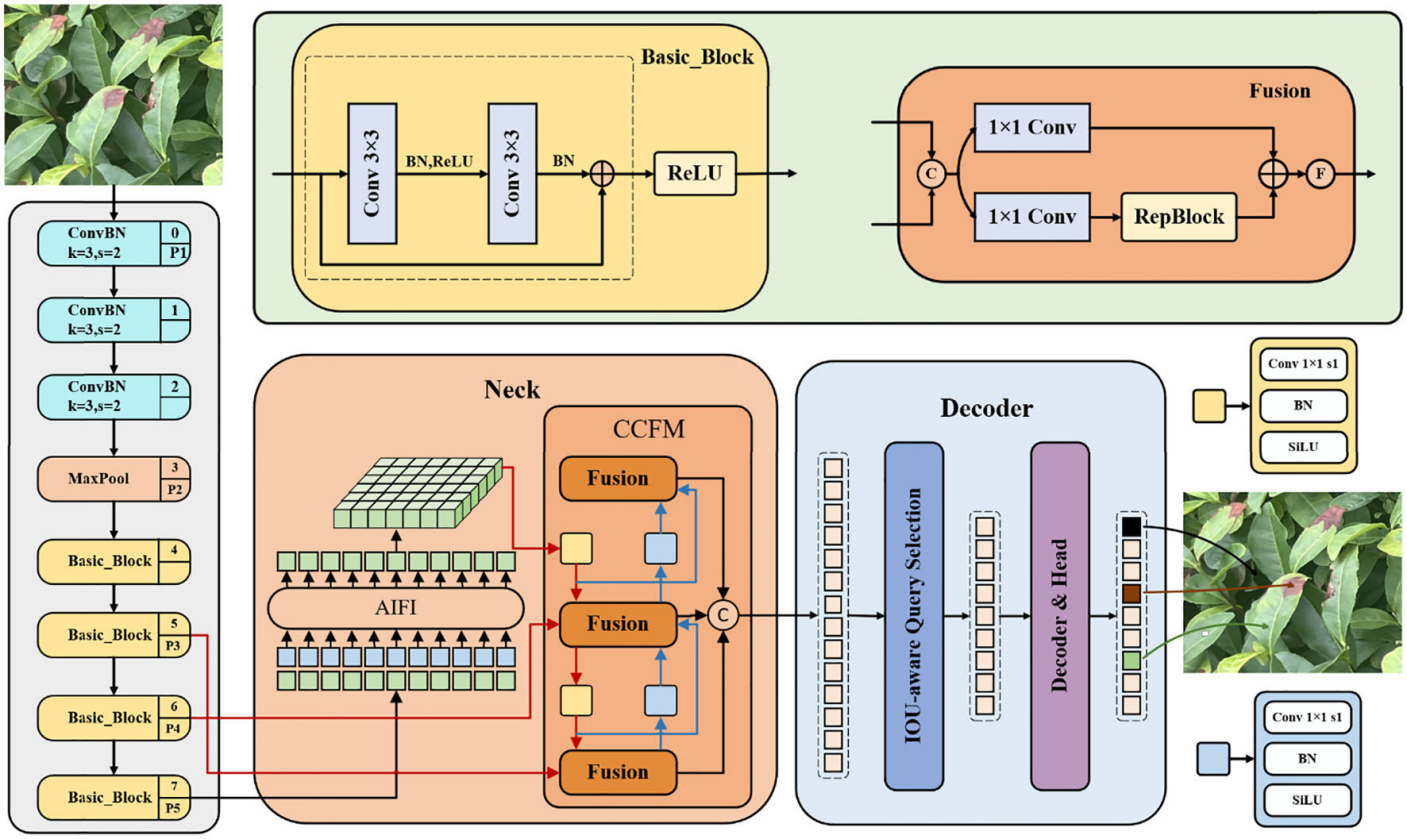
RT-DETR network structure diagram.

In the backbone network, RT-DETR uses convolutional neural networks to extract key features at three different scales, corresponding to outputs with strides of 8, 16, and 32, thereby providing rich feature input for subsequent encoders. The hybrid encoder processes high-level features from the backbone network through the AIFI, which significantly reduces the computational overhead and improves the processing speed. In addition, the encoder utilizes the CCFF to integrate and interact with multi-scale features, fusing the high-level feature maps processed by AIFI with the low-level feature maps. This ensures the model can efficiently detect target objects across various scales. In the decoder part, the model generates the initial query from the encoder output features using an uncertainty minimizing query selection mechanism. These queries interact with the encoder feature maps in the decoder through self-attention and cross-attention mechanisms, and are combined with a feed-forward neural network to generate the category of the object. Overall, RT-DETR improves detection efficiency while retaining the speed advantage of the YOLO series, providing a promising solution for real-time object detection in practical applications. The specific structure of RT-DETR is shown in **Figure 3**.

#### 2.2.2 WMC-RTDETR model architecture

**Figure 4:**
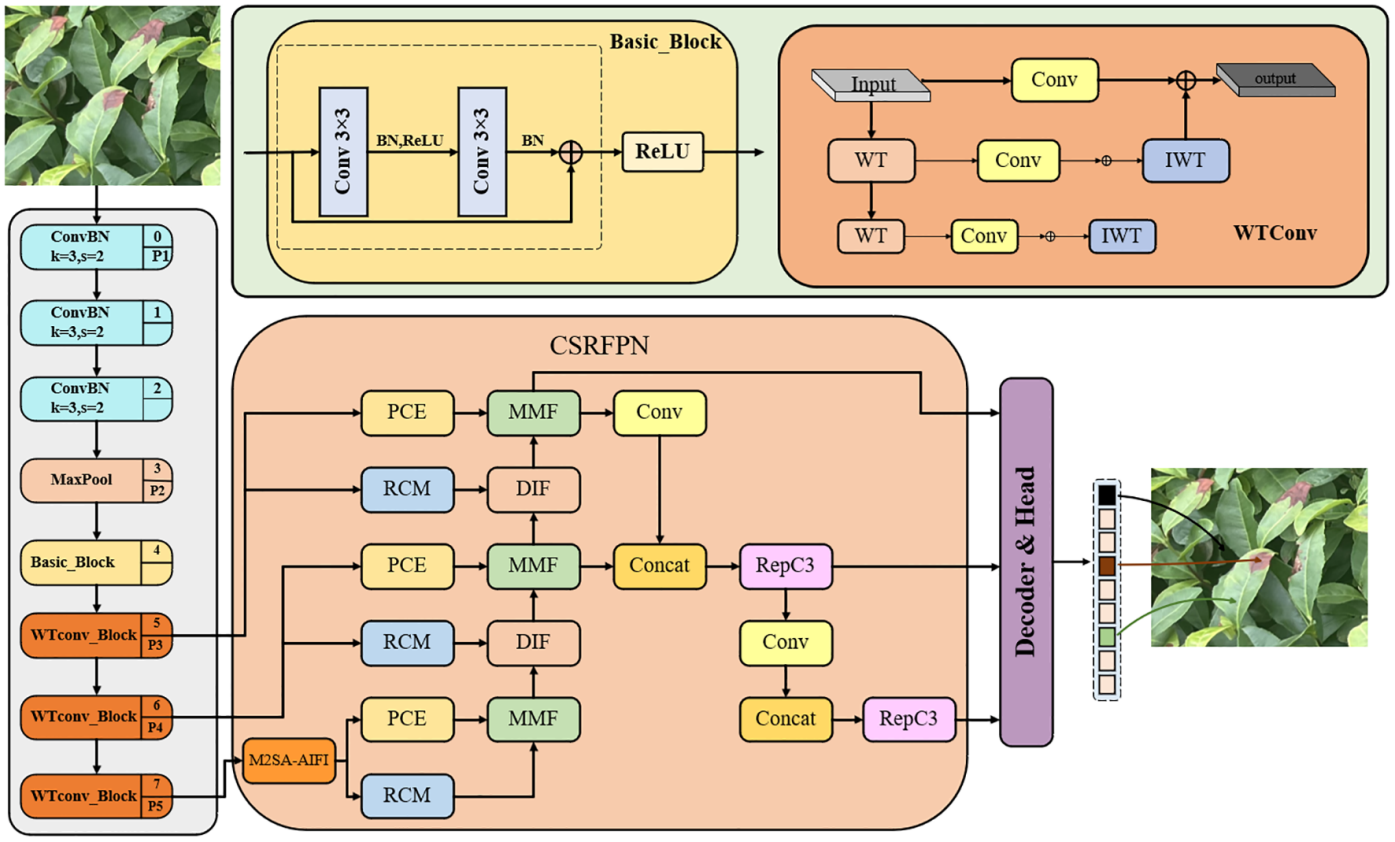
The structure diagram of WMC-RTDETR.

In order to improve the detection capability of tea pests and diseases, especially when there is severe occlusion between targets, we constructed a model based on the RT-DETR network and proposed a new model WMC-RTDETR. This model replaces traditional convolution with WTConv and integrates it into the backbone network of RT-DETR (Finder et al., 2024). The introduction of WTConv extends the receptive field effectively, enhances the capture of low-frequency shape features, and significantly reduces both the number of parameters and computational overhead. Additionally, the M2SA module mechanism was introduced into the AIFI module (Wu et al., 2023). M2SA achieves cross-scale global feature fusion through multi-scale self-attention methods, overcoming the limitations of traditional attention mechanisms in small target detection and multi-scale scenarios, and significantly improves the detection capabilities of RT-DETR in different target scales. In addition, the CSRFPN is integrated into the CCFF module to use context information to guide the spatial feature reconstruction of the target, effectively alleviating the detection difficulties caused by complex background and target occlusion. As a result, the robustness of RT-DETR in intricate background scenarios is significantly enhanced (Ni et al., 2024). Experimental results show that the proposed improvements significantly reduce the number of parameters and floating-point operations (FLOPs) of RT-DETR, while improving the efficiency of real-time detection. The improved model structure is shown in **Figure 4**.

#### 2.2.3 Wavelet transform convolution

To minimize the introduction of excessive parameters and floating-point operations in image recognition tasks, we selected ResNet18 as the baseline of the backbone network (He et al., 2016). Traditional convolutional neural networks usually rely on fixed-size receptive fields and convolution kernels to extract features. Although the receptive field can be expanded by stacking convolutional layers and pooling operations, it is difficult to capture both global and local information in a single layer. Such limitations are particularly evident in multi-scale feature extraction, especially when processing images containing objects of different sizes or complex backgrounds (Gong et al., 2024). Therefore, we introduce WTConv into the backbone network of RT-DETR (Finder et al., 2024). Unlike traditional convolution, which only operates in the spatial domain and lacks frequency domain decomposition capabilities, WTConv use wavelet transform to decompose the input signal into low-frequency and high-frequency components. The low-frequency components capture global structural information, while the high-frequency components extract detail features. This dual capability enhances the multi-scale detection capability of the model. This feature of simultaneous feature extraction in the time domain and frequency domain makes WTConv excellent in capturing subtle features and suppressing noise. WTConv enhances the target detection performance of RT-DETR in complex and noisy environments, improving robustness without a substantial increase in computational cost.

**Figure 5:**
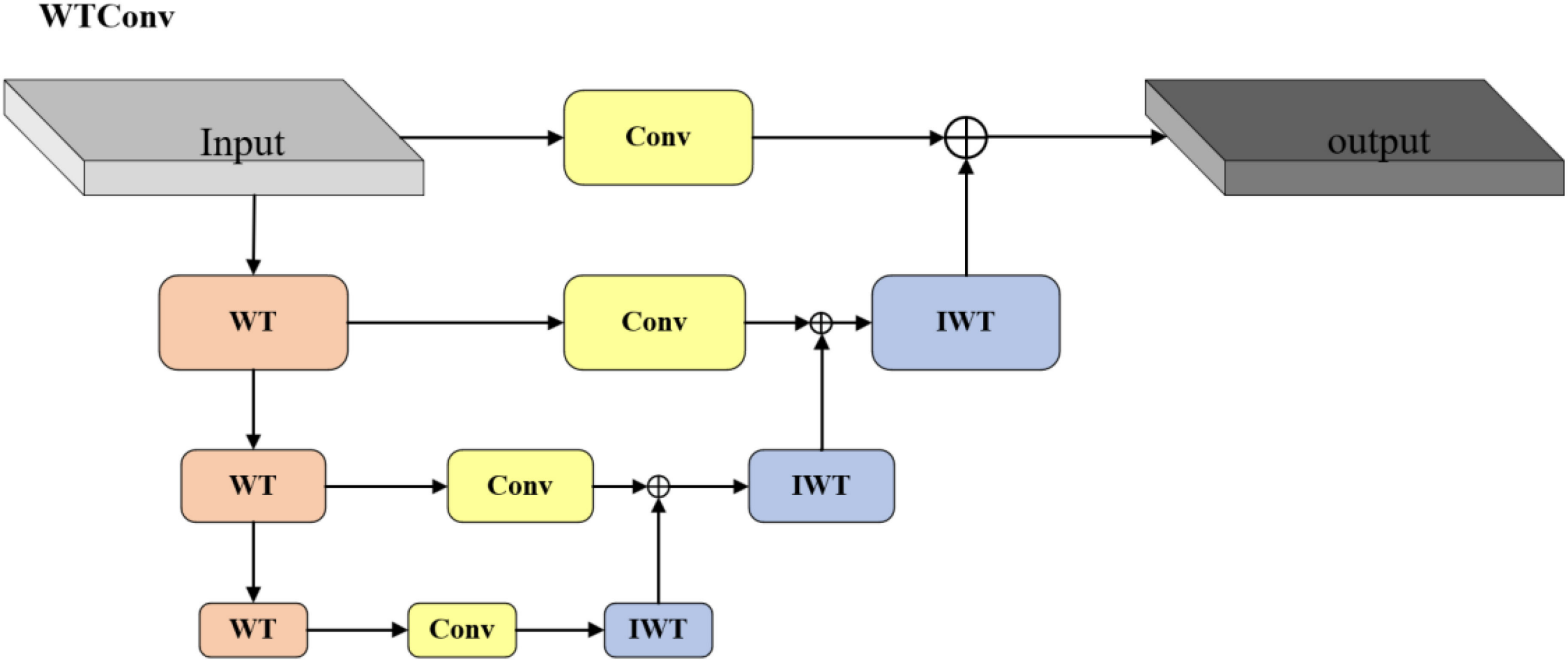
The structure of WTConv.


The FLOPs of WTConv mainly consist of three parts: wavelet decomposition, convolution operations on each frequency band, and inverse wavelet transform. The specific process is shown in **Figure 5**. We assume that the input feature map size is H×W , the convolution kernel size is K×K , the number of input channels is $C_{\text{in}}$ , and the number of output channels is $C_{\text{out}}$ . Wavelet Transform (WT) decomposes the input feature map into different frequency bands: low-frequency (LL) and high-frequency (LH, HL, HH) components. Through decomposition, the spatial dimension of each frequency band is reduced to 1/2 of the original size (assuming the use of Haar wavelet), so the size of each frequency band is H/2×W/2 . The FLOPs of wavelet decomposition can be expressed as Equation 1:

(1) $$FLOPs_{WT}=C_{\text{in}}\times \sum_{\text{i}=0}^{\ell -1}4\times \frac{H\times W}{2^{\text{i}}}$$

where ℓ is the number of wavelet decomposition layers, and each layer further decomposes the low-frequency components, further reducing the spatial resolution. Since the size of the feature map after decomposition is reduced, the computational growth for this operation is gradual.

After decomposition, convolution with a small kernel (e.g., k×k ) is performed on each frequency band. The amount of convolution calculation for each frequency band can be expressed as Equation 2:


(2) $$FLOPs_{WTC\text{onv}}=\sum_{\text{i}=1}^{\ell }\left(\frac{H*W}{2^{i}}\times k^{2}\times 4\times C_{in}\times C_{out}\right)$$

The reduction in resolution by a factor of $2^{i}$ and convolution on decomposed frequency bands significantly lowers computational effort compared to applying large kernels on the original spatial dimensions.

After completing the convolution on all frequency bands, WTConv uses the inverse wavelet transform (IWT) to reconstruct the output feature map. The computational complexity of the inverse wavelet transform is similar to that of the wavelet decomposition, which can also be expressed as Equation 3:


(3) $$FLOPs_{IWT}=C_{out}\times \sum_{\text{i}=0}^{\ell -1}4\times \frac{H\times W}{2^{\text{i}}}$$

Since the spatial dimension of the feature map of WTConv is reduced after each layer of decomposition, even if the computation on each frequency band is accumulated, the total FLOPs of WTConv still grows slowly. Compared to directly using large convolution kernels on the original feature map, WTConv can achieve a larger receptive field with less floating-point operations.

#### 2.2.4 Multiscale multihead self-attention

**Figure 6:**
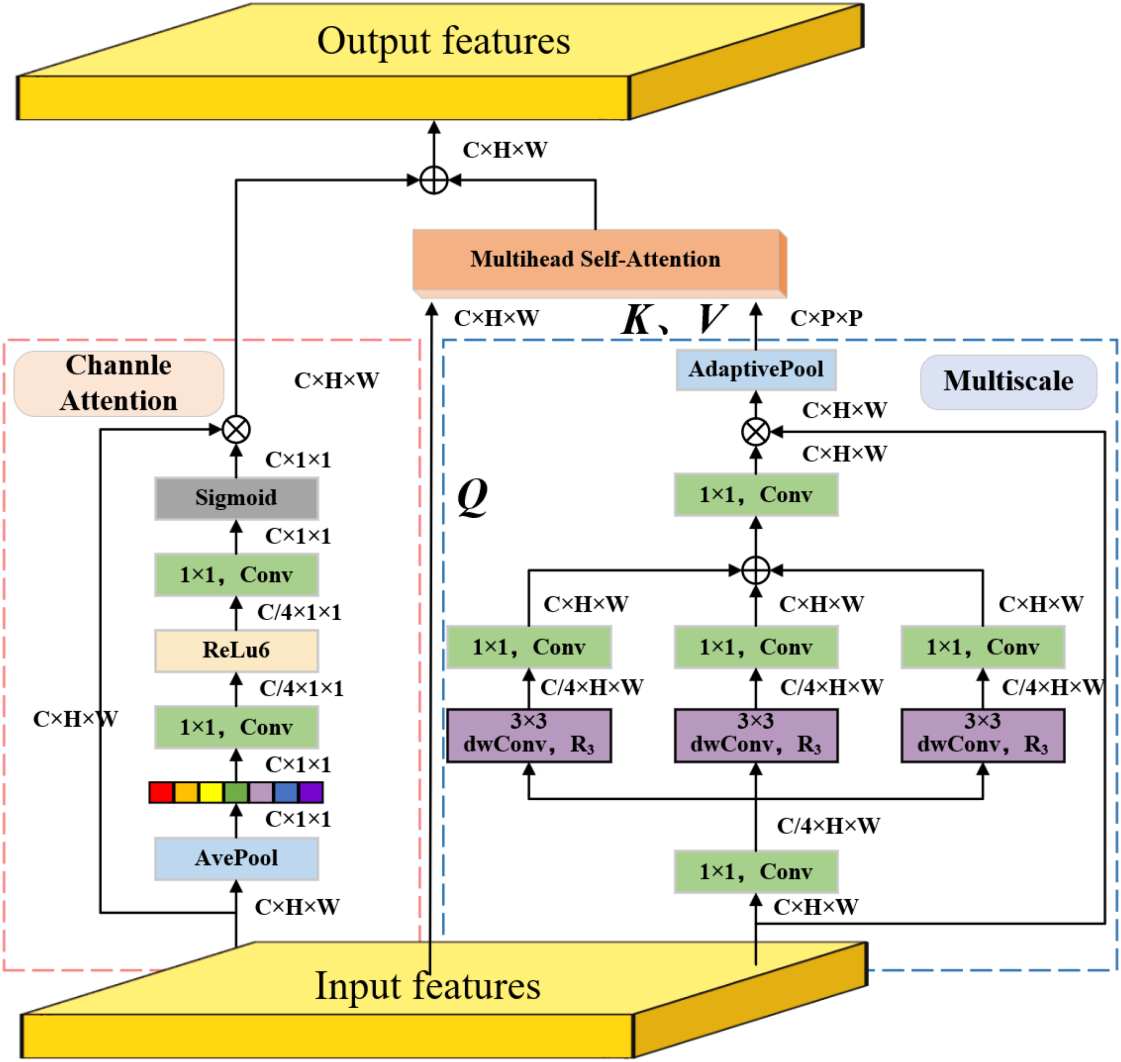
The structure of M2SA.

In RT-DETR, the AIFI module adopts a multiscale multihead self-attention module to achieve adaptive fusion of instance features. However, since it is limited to fixed-scale feature processing, the module struggles effectively capture the multi-scale semantic information between instances (Zhang et al., 2023a). To solve this problem, this study introduced the multiscale multihead self-attention module to enhance the AIFI module’s ability to capture multi-scale features (Wu et al., 2023). M2SA consists of three parts: multi-scale mechanism, multi-head self-attention mechanism and channel attention mechanism. This enables M2SA to not only extract rich global information at different scales, but also optimize the feature weight distribution through channel attention. This significantly improves the ability to model target features in complex scenes. The structure of M2SA is shown in **Figure 6**.

In the multi-scale mechanism, the input feature $X\in {\mathbb{R}}^{C\times H\times W}$ is first reduced to one-fourth of its original size through 1×1 convolution and used as the initial feature. Then multiple depthwise separable atrous convolutions are used to set different atrous rates to generate multi-scale features, as specified in Equation 4:


(4) $$X_{\text{i}}=dwConv_{R_{i}}^{3\times 3}(Conv_{1\times 1}(X)),i=\{1,3,5\}$$

Among them, $dwConv_{R_{i}}^{3\times 3}()$ represents atrous convolutions, and the value of atrous rate can be {1,3,5} , which is used to extract features under different receptive fields. Next, the original number of channels is restored with 1×1 convolution and all scale features are summed and fused, as specified in Equation 5:


(5) $$X_{out}=Conv_{1\times 1}(X_{1}+X_{2}+X_{3})⨀X$$

The fused feature contains multi-scale global context information and is multiplied element-wise ( ⊙ represents element-by-element multiplication) with the input feature X to generate the final multi-scale fused information feature, as specified in Equation 6:


(6) $$X_{msa}=X_{out}⨀X$$

In the multi-head self-attention mechanism, in order to reduce the computational complexity, the downsampled multi-scale features $X_{msa}$ are used to generate K (Key) and V (Value), while the original feature is used for the Q (Query), which is specifically expressed as Equation 7:


(7) $$\left(Q,K,V)=(XW^{q},X_{msa}W^{k},X_{msa}W^{v}\right)$$

where $W^{q},W^{k},W^{v}$ are the linear transformation matrixs. Then, Q,K,V are sent to the multi-head self-attention module to calculate the self-attention features, as specified in Equation 8:


(8) $$Attention=Soft\max(\frac{Q\times K^{T}}{\sqrt{d_{k}}})\times V$$

where $\sqrt{d_{k}}$ is the channel dimension of K . This process captures long-range dependencies in space by taking a weighted average at each position in space.

Nevertheless, in the channel attention mechanism, the input feature X is globally averaged and pooled to generate a C×1×1 channel descriptor $X_{p}$ to better fit the 2-D image structure, as specified in Equation 9:


(9) $$X_{p}=AvgPool(X)$$

Then, the channel descriptor $X_{p}$ passes through a series of 1×1 convolution operations. First, the channel dimension is reduced through a 1×1 convolution, then a nonlinear transformation is performed through an activation function (ReLU6), and finally another 1×1 convolution is performed to restore the original number of channels. Next, the Sigmoid activation function is used to generate the channel attention weight distribution $Attention_{c}$ . Then, the channel attention weight distribution is multiplied by the input feature X by element-by-element multiplication to obtain the weighted feature map $X_{ca}$ , as described in Equations 10–12:


(10) Xc=ReLu6(Conv1×1(Xp))

(11) $$Attention_{c}=Sigmoid(Conv_{1\times 1}(X_{c}))$$

(12) $$X_{ca}=Attention_{c}⊙X$$

Finally, the features of the above three branches are fused to obtain the final output features, as defined in Equation 13:


(13) $$X_{AIFI}=X_{msa}+Attention+X_{ca}$$

#### 2.2.5 Context-guided spatial feature reconstruction feature pyramid network

**Figure 7:**
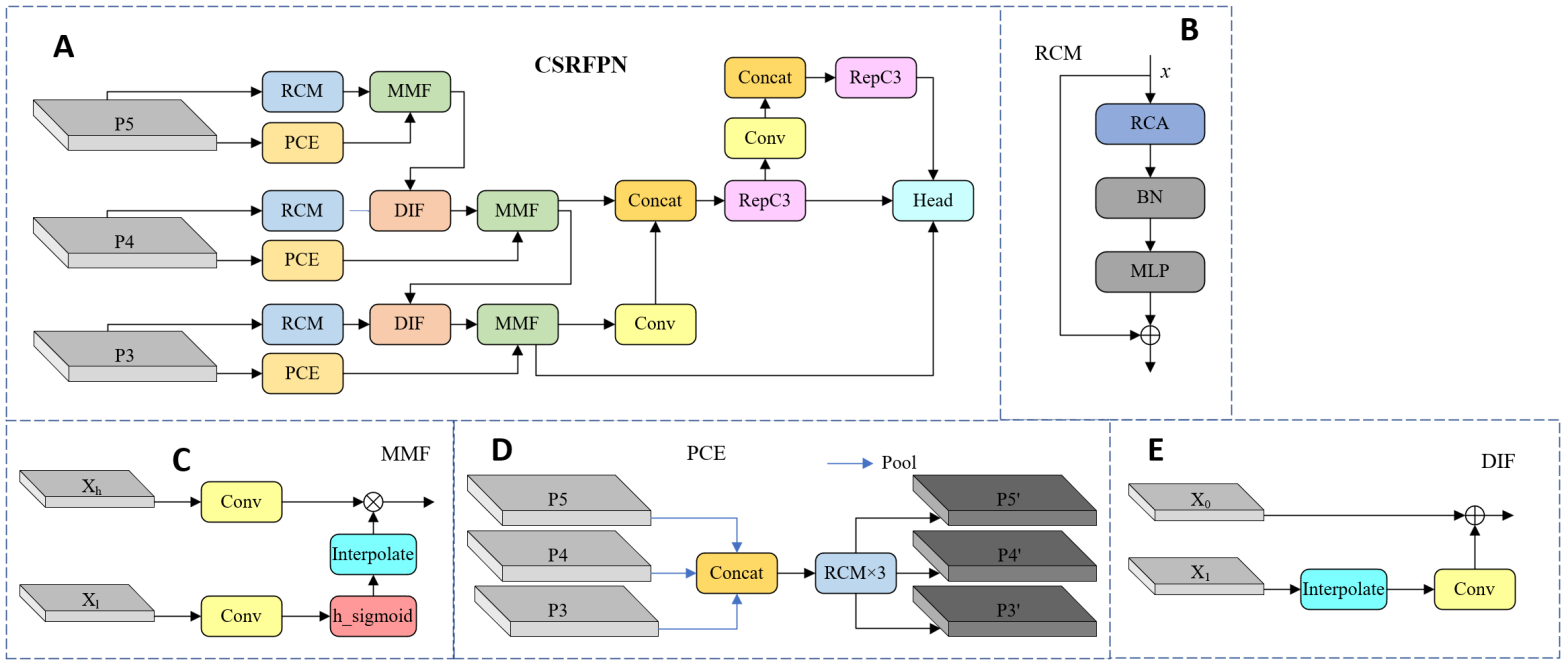
The structure diagram of CSRFPN. **(A)** CSRFPN, **(B)** RCM, **(C)** MMF, **(D)** PCE, **(E)** DIF.

In the RT-DETR model, the CCFF Module is responsible for the fusion of multi-scale features, aiming to integrate features from different scales. However, the feature fusion methods currently used by CCFF mainly rely on simple feature superposition or layer-by-layer fusion, which is difficult to effectively capture multi-scale contextual information, especially when targets vary significantly in size. To this end, this paper draws on the design concept of CGRSeg and introduces two key modules (Ni et al., 2024): Rectangular Self-Calibration Module (RCM) and Pyramid Context Extraction Module (PCE) (Wang et al., 2024b). We replace the original element-wise addition and multiplication with the independently developed Dynamic Interpolation Fusion (DIF) and Multi-Feature Fusion (MFF) modules, enhancing multi-scale feature representation and improving target recognition in complex backgrounds. The specific structure of CSRFPN is shown in **Figure 7**.

##### 2.2.5.1 Rectangular self-calibration module

First, through horizontal and vertical pooling operations, the global context information of the input feature map in the horizontal and vertical directions is extracted respectively. This operation can effectively model the key features of the rectangular area. If the input feature map is $P\in {\mathbb{R}}^{C\times H\times W}$ , after horizontal pooling and vertical pooling, an axial feature vector will be obtained, which are $H_{p}\in {\mathbb{R}}^{C\times 1\times W},V_{p}\in {\mathbb{R}}^{C\times H\times 1}$ . Then, the two axial vectors are fused through broadcast addition to form a rectangular attention region.

Next, in order to make the generated rectangular attention area closer to the foreground features, RCM uses two large kernel convolutions to adjust in the horizontal and vertical directions respectively. First, convolution is performed in the horizontal direction, then activated by ReLU nonlinearity, and the same operation is applied in the vertical direction to form a self-calibrated foreground area, as described in Equation 14:


(14) $$\xi c(y)=\delta (\psi_{k\times 1}(\phi (\psi_{1\times k}(H_{p}⊕V_{p}))))$$

where, ψ represents large-kernel strip convolution, k represents the kernel size of the strip convolution, ϕ represents the Batch Normalization followed by the ReLU function, and δ represents the Sigmoid function.

Finally, the self-calibrated rectangular attention features are fused with the input feature map (element-wise multiplication) to enhance the feature expression of the foreground area, as described in Equation 15:


(15) $$\xi_{F}(x,y)=\psi_{3\times 3}(x)⊙y$$

Among them, $\psi_{3\times 3}$ represents the deep-wise convolution with kernel 3×3, y is the attention feature obtained in the previous step, and ⊙ represents element-by-element multiplication.

##### 2.2.5.2 Pyramid context extraction module

This module first downsamples the input multi-scale feature map to construct a pyramid structure. In RT-DETR, the input feature maps come from different levels of the feature extraction network, denoted as $P_{3},P_{4},P_{5}$ . After constructing the pyramid structure, the PCE module further extracts multi-scale contextual information by stacking multiple RCM modules. The RCM module captures global context and performs spatial reconstruction on each layer of feature maps to ensure that features at each scale in the pyramid structure can benefit from global information. The application of multi-layer RCM improves the expressiveness of features at each scale and enhances the context-awareness of the model. Subsequently, the contextual information of different scales is integrated to form the final multi-scale feature output P . These features contain rich contextual information at the spatial and semantic levels, providing multi-scale support for subsequent detection and segmentation tasks. This process can be expressed as Equation 16:


(16) $$P=RCM(AP(P_{3},8),AP(P_{4},4),AP(P_{5},2))$$

Among them, AP(P, x) means average pooling of feature P with factor x .

##### 2.2.5.3 Dynamic interpolation fusion

The DIF module is mainly used to integrate feature maps with different channel numbers and spatial resolutions. This module achieves alignment of scale and channel through interpolation and convolution operations, subsequently completes feature fusion through element-by-element addition. During forward propagation, for the input feature map $X=(X_{0},X_{1})$ , the low-resolution feature map $X_{1}$ is first adjusted to the same spatial resolution as $X_{0}$ through bilinear interpolation. Then, the interpolated $X_{1}$ is adjusted to have the same number of channels as $X_{0}$ through a 1×1 convolution to obtain $X_{1}^{'}$ , as shown in Equation 17:


(17) $$X_{1}^{'}=Conv_{1\times 1}(Interpolate(X_{1}))$$

Among them, $X_{0}$ and $X_{1}$ represent feature maps of two different scales, Interpolate is a bilinear interpolation algorithm, and $Conv_{1\times 1}$ is a 1×1 convolution. Add the result to $X_{0}$ element by element to get the fused feature map, as shown in Equation 18:


(18) $$X_{out}=X_{1}^{'}⊕X_{0}$$

##### 2.2.5.4 Multi-feature fusion

The MFF module uses the adaptive activation signal of low-resolution maps to guide high-resolution feature maps, effectively fusing detail and context, ideal for adaptive fusion across scales. For input feature maps $\left(X_{h},X_{l}\right)$ , both are first convolved, and then the low-resolution feature map after convolution is normalized using the activation function h_sigmoid to control the range of the signal, and $L(X_{l})$ is obtained as Equation 19:


(19) $$L(X_{l})=h\_sigmoid((Conv(X_{l})))$$

Among them, $X_{l}$ is a low-resolution feature map, and $X_{h}$ is a high-resolution feature map. Then bilinear interpolation is applied to adjust $L(X_{l})$ to the resolution of $X_{h}$ so that the two can be multiplied element by element. The overall formula can be summarized as Equation 20:


(20) $$X_{out}=Interpolate(L(X_{l}))⊗Conv(X_{h})$$

## 3 Evaluation indicators

This study evaluated the algorithm performance by comparing the image detection differences before and after the model improvements under the same experimental settings. This study uses precision (P), recall (R), mean average precision (mAP), F1 score, GFLOPs and frames per second (FPS) as evaluation criteria.

Precision refers to the proportion of correctly predicted positive classes to all predicted positive classes. It reflects the reliability of model predictions. If the precision is high, it means that the model is more reliable in predicting the correctness. The specific formula can be expressed as Equation 21:


(21) $$Precision=\frac{TP}{TP+FP}$$

Among them, TP (True Positive) indicates the number of correctly detected targets, while FP (False Positive) indicates the number of targets that were incorrectly detected, that is, the background or other objects are mistakenly identified as targets.

Recall refers to the proportion of targets successfully detected by the model to all actual targets. Recall reflects the coverage ability of the model. If the recall rate is high, it means that the model can identify most of the targets. The specific formula can be expressed as Equation 22:


(22) Recall=TPTP+FN

Among them, FN (False Negative) represents the number of targets that were missed, that is, they are actually targets but are not detected by the model.

Average Precision (AP) is the area under the PR curve of a category within all predicted pictures. The Mean Average Precision (mAP) averages these APs across different categories to evaluate the overall performance of the object detection model. It is a comprehensive indicator suitable for multi-category object detection tasks. The specific formula can be expressed as Equations 23, 24:


(23) $$AP=\int_{0}^{1}P(r)dr$$

(24) $$mAP=\frac{\sum_{j=1}^{N}AP(j)}{N}$$

where N is the number of all categories.

The F1 score is the harmonic mean of Precision and Recall, and is an indicator that comprehensively reflects the accuracy and comprehensiveness of detection. The F1 score ranges from 0 to 1. With values closer to 1 indicating superior model performance in terms of accuracy and comprehensiveness, as shown in Equation 25.


(25) $$F1=2*\frac{P*R}{P+R}$$

GFLOP (Giga Floating Point Operations Per Second) is an indicator to measure the complexity and computational complexity of a model. It indicates the number of floating-point operations required for a model to perform one inference, measured in billions of operations. Models with high GFLOPs are usually more complex, computationally intensive, and require more computing resources.

FPS (Frames Per Second) refers to the number of image frames that the model can process per second in a test environment, it is an important indicator for measuring the speed of model reasoning, as shown in Equation 26.


(26) $$FPS=\frac{1}{t_{avg}}$$

where $t_{avg}$ represents the average inference time.

## 4 Results analysis

### 4.1 Experimental configuration

All training and evaluation in this paper are completed under the same parameter setting. The specific environment is shown in **Table 1**. The batch size is set to 4, the number of training rounds is 300 epochs, the learning rate is set to 1×10−4, and the image size of 640×640 is selected for the experiment. The loss function during training is uniformly set to GIOU.

**Table 1:** Hardware configuration and model parameters.

Environment	Disposition
Operating system	Linux
CPU	Intel(R) Xeon(R) Gold 5418Y * 12 core
GPU	NVIDIA RTX 4090
Pytorch	2.2.2
CUDA	12.1
Python	3.10
optimizer	AdamW

### 4.2 Backbone network comparative experiment

In order to verify the superior performance of Wavelet Transform Convolution, we selected advanced convolutional networks in recent years as a comparison, and the specific results are shown in **Table 2**.

**Table 2:** Comparative experiments of different backbone networks.

Backbone	Precision (%)	Recall (%)	mAP50 (%)	mAP50:95 (%)	Params (M)	GFLOPS (G)
Basic	96.8	99	96.2	69.9	19.87	56.9
AKConv	93.7	99	95.9	75.8	15.6	48.5
DCNv2	93.4	99	94.5	74.5	20.22	53.7
PConv	94.1	99	95.8	78	14.1	43.2
StarConv	94.6	99	97.6	80	22.17	60.5
WTConv	94.6	99	97.4	80.9	13.95	42.6

From **Table 2**, it is easy to see that compared with AKConv (Zhang et al., 2023b), Deformable Convolution v2 (Zhu et al., 2019), PConv (Chen et al., 2023), and StarConv (Ma et al., 2024), our WTConv effectively reduces the amount of computation while maximizing the accuracy. Although StarConv performs well on the mAP50 metric, the number of parameters and floating-point computation are significantly increased compared to the original backbone network, which does not satisfy the requirement of lightweighting. In summary, WTConv offers the best balance of accuracy and lightness.

### 4.3 Ablation experiment

To verify the effectiveness of each improved module, we use the original RT-DETR as the base network for ablation experiments. The detailed experimental results are listed in detail in **Table 3**.

**Table 3:** Ablation results.

Model	WTConv	M2SA	CGRFPN	Params (M)	GFLOPS (G)	Size (M)	Fps (f/s)	mAP50 (%)
Base				19.87	56.9	40.5	23.1	96.2
1	✓			13.95	42.6	27	23.8	97.4
		✓		19.9	57.1	40.6	71.9	97.6
2	✓	✓		13.31	40.3	28.9	54.8	97.6
			✓	19.23	48.2	39	25.9	97.5
Ours	✓	✓	✓	12.82	33.9	27.5	58.8	97.7

The results of the ablation study presented in **Table 3** demonstrate that the original RT-DETR model performs at 96.2% on the mAP50 metric. Based on the original model, Model 1 effectively enhances the image feature extraction capability by incorporating WTConv, increases mAP50 to 97.4%, and reduces the number of model parameters. Model 2 adds the M2SA module further, which slightly improves the mAP50 and greatly improves the Fps of the model. Finally, our model integrates the three improved modules, with an mAP50 of 97.7% and an FPS of 58.8, while the number of parameters, floating-point operations, and model size are reduced by 35.48%, 40.42%, and 32.09%, respectively. The data shows that this comprehensive improvement strategy not only improve the detection performance, but also effectively reduce the model size and improve the processing speed.

### 4.4 Comparative experiment

To further verify the effectiveness of the proposed model, we compare the various performance indicators of the model with other models, including Faster R-CNN (Ren et al., 2016), SSD (Liu et al., 2016), YOLOv5 (Jocher et al., 2022), YOLOv6 (Li et al., 2022), YOLOv8, YOLOv9, YOLOv10 (Wang et al., 2024a), YOLOv11, as shown in **Table 4**.

**Table 4:** Comparison of results.

Model	Precision (%)	Recall (%)	mAP50 (%)	mAP50:95 (%)	F1 (%)	Weight (MB)
Faster R-CNN	16.77	30.42	10.44	5.8	21	108
SSD	55.55	4.1	10.22	4.2	7.5	91.1
YOLOv5n	94.3	97	94.3	76.7	94	5.3
YOLOv6n	95.8	97	95	78.3	94	8.7
YOLOv8n	95.5	98	95.7	78.4	95	6.3
YOLOv9s	96.9	98	96.3	81.9	96	15.3
YOLOv10n	96.6	97	94.2	75.1	91	6.5
YOLOv11n	96.7	92.9	96	76.7	95	5.5
RT-DETR	96.8	99	96.2	69.9	94	40.5
RT-DETR-R34	95.7	99	97.1	82.8	96	63
RT-DETR-R50	96.7	99	97.7	82.8	97	86.1
WMC-RTDETR	96.8	99	97.7	83.1	96	27.5

The improved model has 3.4%, 2.7%, 2%, 1.4%, 3.5%, and 1.7% higher map50 than YOLOv5, YOLOv6, YOLOv8, YOLOv9, YOLOv10, and YOLOv11, respectively. Compared with the original RT-DETR model, mAP50 and mAP50:95 are improved by 1.5% and 13.2%, respectively. Therefore, compared with other object detection networks, our model shows superior performance in detecting tea pests and diseases in natural environments.

### 4.5 Visual comparison of detection results

**Figure 8:**
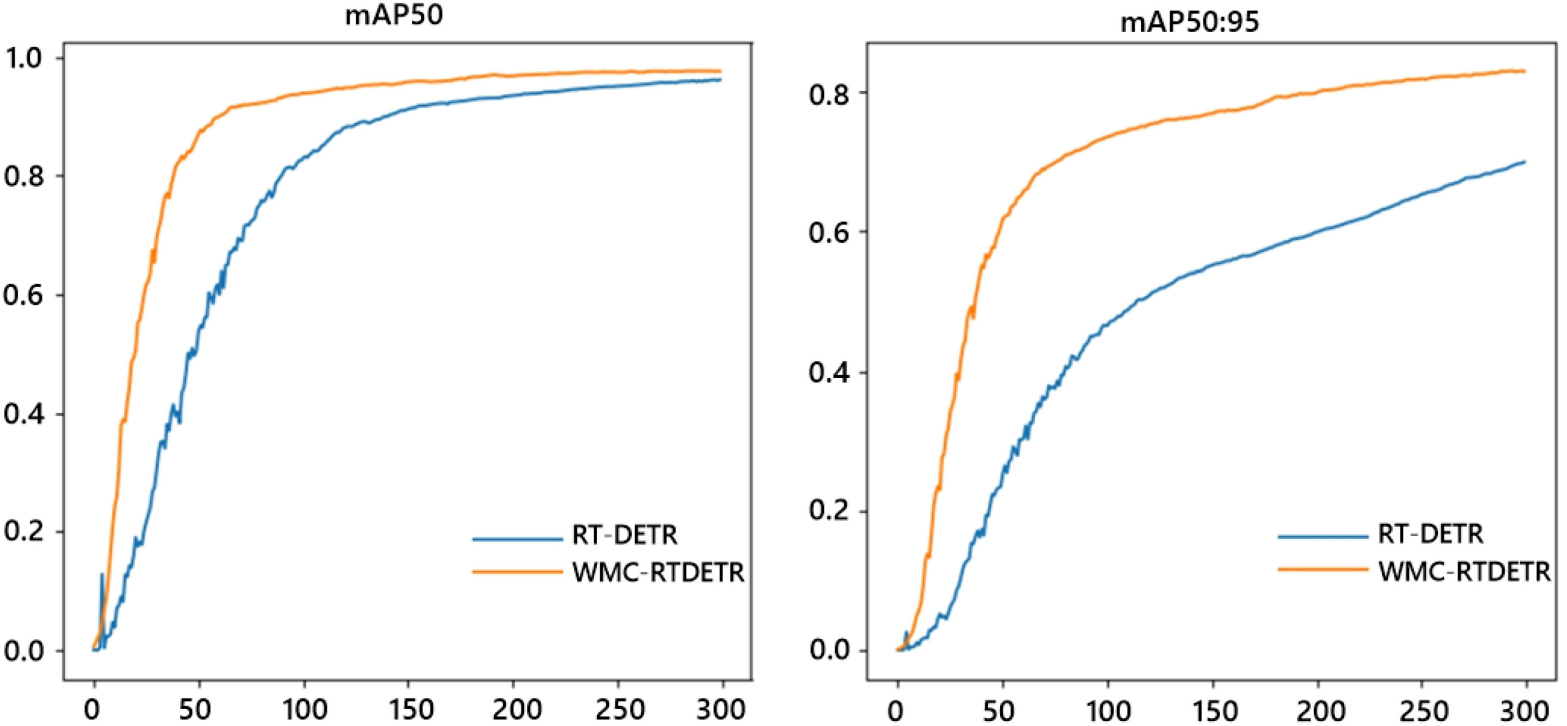
Comparison of mean average precision between base model and ours.

During the training process of the original model and the WMC-RTDETR model, identical datasets and parameter settings are utilized. Based on the log files generated during training, the mAP50 and mAP50:95 training curves of the original model and the improved model during training are plotted, as shown in **Figure 8**. The blue curve represents the basic RT-DETR model, and the orange curve represents our improved WMC-RTDETR model. As illustrated in the figure, the improved model exceeds the original model in terms of mAP50 and mAP50:95 indicators at all stages of training, indicating that WMC-RTDETR has higher accuracy and effectiveness in tea pest and disease detection.

**Figure 9:**
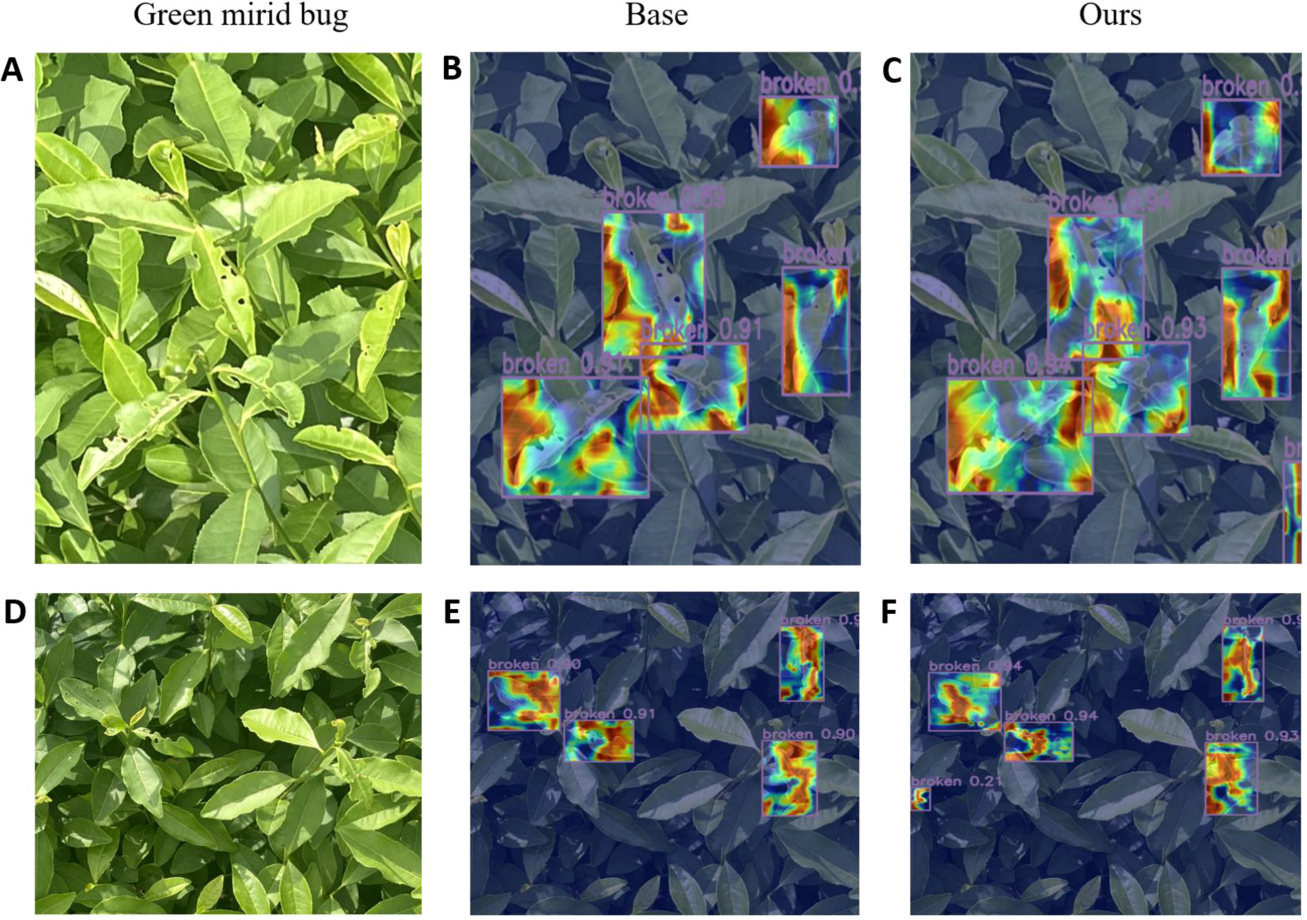
Visualization of heat map of green mirid bug. **(A, D)** images of green mirid bug, **(B, E)** heatmaps of RT-DETR, and **(C, F)** heatmaps of WMC-RTDETR.

**Figure 10:**
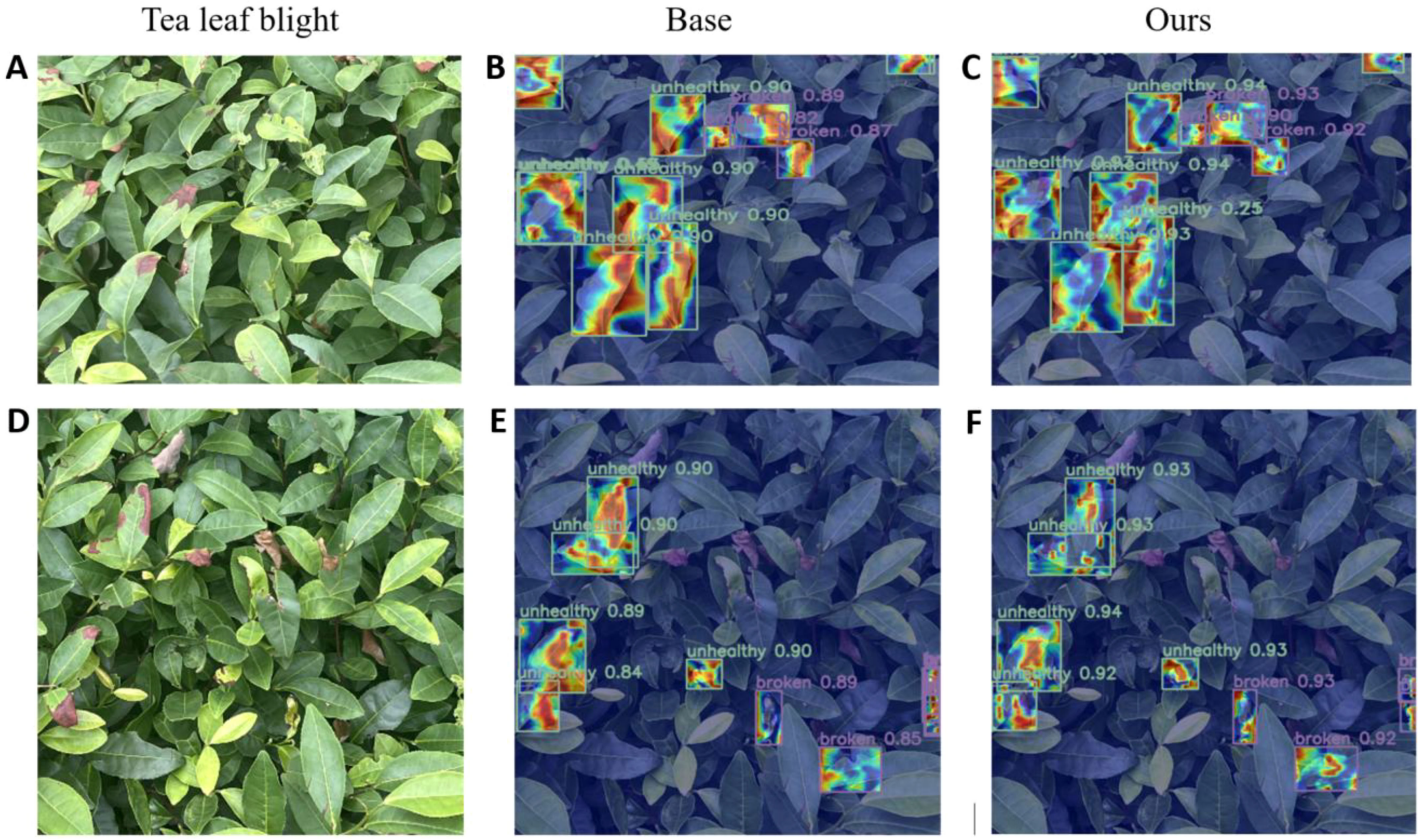
Visualization of heat map of tea leaf blight. **(A, D)** images of tea leaf blight, **(B, E)** heatmaps of RT-DETR, and **(C, F)** heatmaps of WMC-RTDETR.

In order to more clearly compare the performance differences of the models in the object detection task, we used the EigenCAM heat map method to perform a visual analysis of the two tea pests and diseases detected and generated heat maps (Muhammad and Yeasin, 2020), as shown in **Figures 9** and **10**.

The above heat map uses a gradient color scale (Jet palette) to visualize the strong focus of the model on the characteristics of the infested area. Specifically, the red area indicates the high response area of the model, which the model considers to contain key features (e.g., disease areas or pest morphology) with the highest level of confidence. Yellow to green regions indicate moderate response strength, corresponding to target edges or secondary features in the background that are relevant to the target. Blue areas indicate low or no response and represent background or irrelevant areas not attended to by the model.

The visualization results show that the basic model’s feature extraction is limited in the target area, with a small high-response heatmap, leading to missed or false detections, particularly in complex backgrounds. At the same time, the confidence of the basic model is generally low, and it pays less attention to the edge area of ​​the target, indicating that it is insufficiently sensitive to detailed features.

In comparison, our improved model shows significant advantages. In the heat map, the high-response area covers the key feature area of ​​the target and shows stronger adaptability in edge areas and complex background scenes. This shows that the improved model can capture target features more comprehensively and effectively reduce missed detections. Meanwhile, from the perspective of confidence comparison, the confidence level of the improved model in classification tasks is generally higher than that of the basic model, which further proves its classification accuracy and robustness.

### 4.6 Detection system based on embedded raspberry Pi

**Figure 11:**
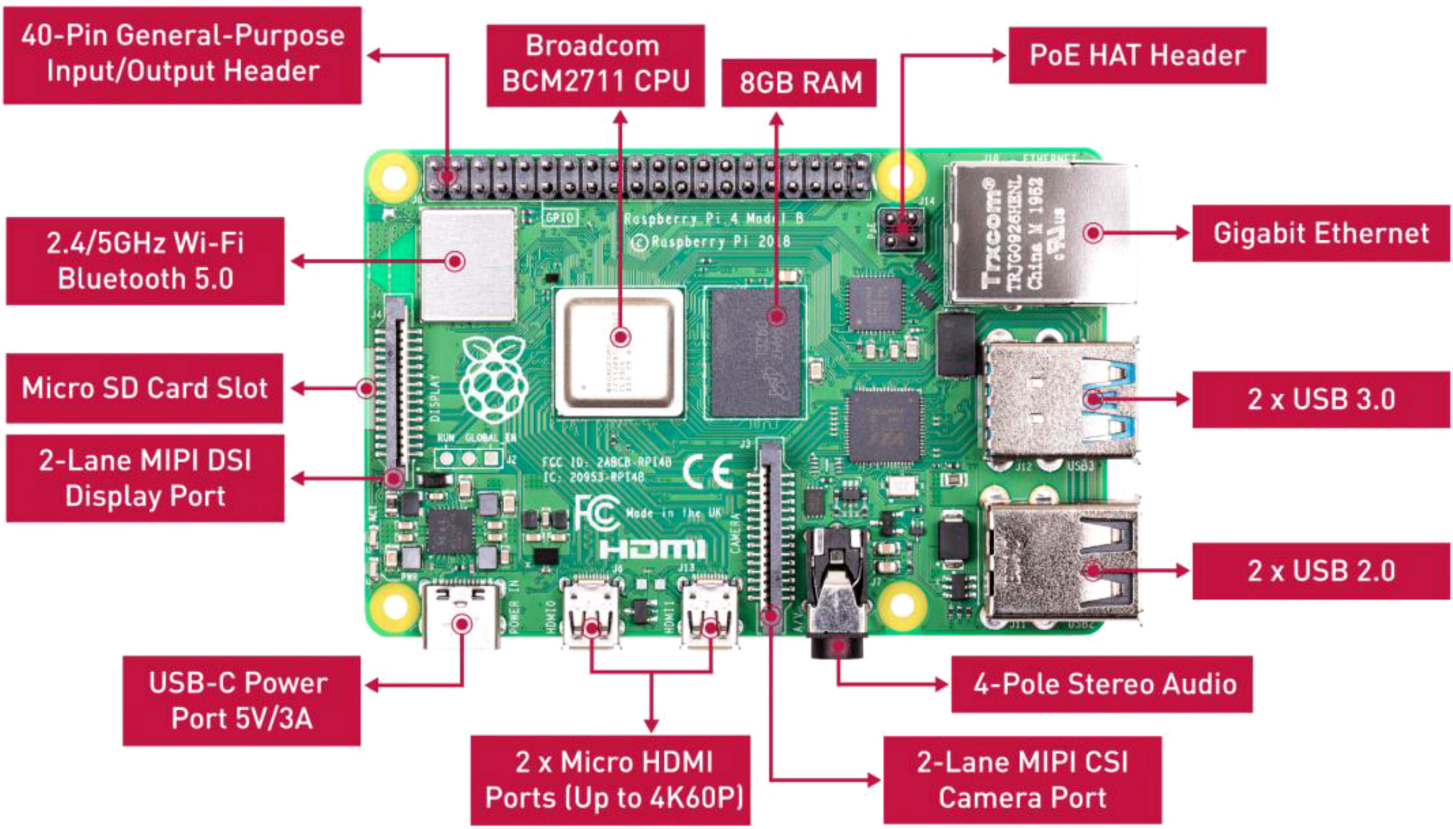
Raspberry Pi 4B physical image.

With the continuous improvement in the performance of small embedded platforms, they have the ability to support some deep learning-based target detection technologies. We will deploy the independently improved lightweight algorithm WMC-RTDETR on the Raspberry Pi to detect tea pests and diseases, in order to achieve efficient transplantation and wide application of the algorithm on low-cost embedded hardware. As a high-performance ARM architecture single-board computer, Raspberry Pi 4B, as shown in **Figures 11**. The specific hardware parameters are shown in **Table 5**.

**Table 5:** Raspberry Pi 4B hardware parameters.

Name	Raspberry Pi 4B
SOC	Broadcom BCM2711
CPU	64-bit 1.5GHz quad-core
GPU	500MHz VideoCore VI
Memory	1-8GB DDR4
Maximum resolution	4K 60Hz+1080p or 2*4K 30Hz
USB port	2 USB3.0/2 USB2.0
Charging port	USB Type-C
Wired network	Gigabit Ethernet
Wireless	802.11ac (2.4/5G)
Power demand	3A,5V

#### 4.6.1 Model deployment

**Figure 12:**
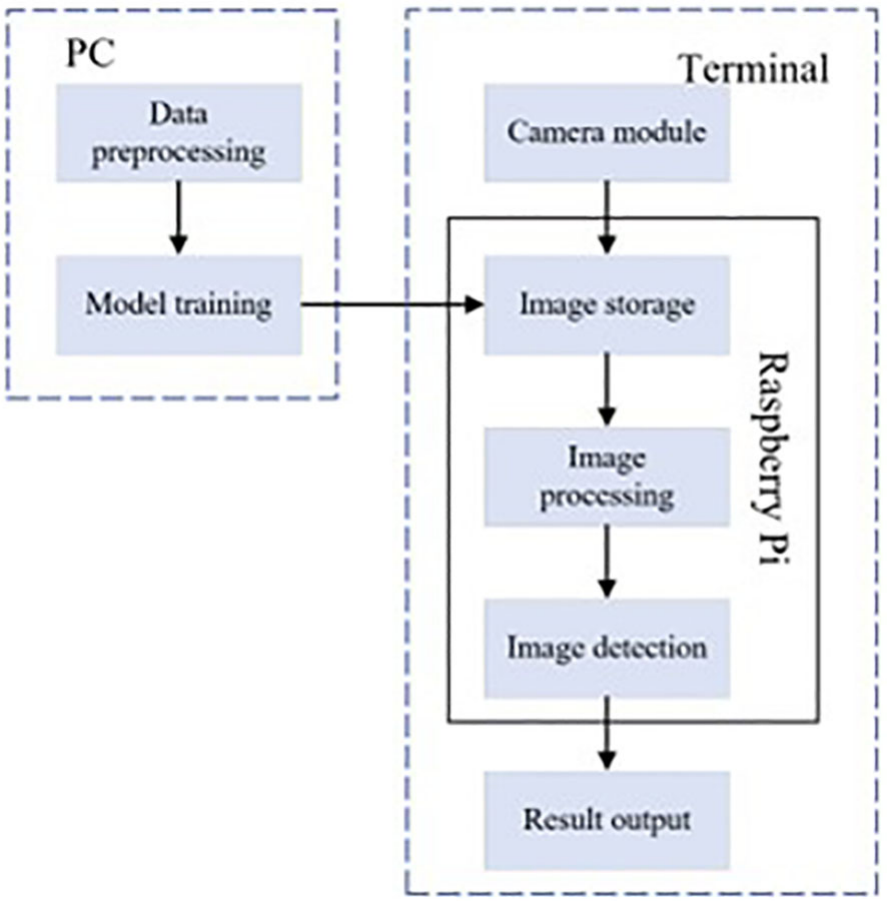
Detection system integration flow chart.

For the manually labeled tea pest and disease dataset, this study trained based on RT-DETR model and further improved the proposed WMC-RTDETR tea pest and disease detection algorithm. The optimized model is deployed to the Raspberry Pi 4B platform to validate the effectiveness and platform adaptability of the algorithm in this paper. The specific process is shown in **Figure 12** (Li, 2023). The experimental results show that the improved model is able to display the detection parameters in real time on the Raspberry Pi platform and accurately recognize various tea pest and disease information, which verifies its efficiency and practicality on low-cost embedded hardware.

#### 4.6.2 Test results analysis

In order to verify the application performance of the WMC-RTDETR tea pest and disease detection algorithm in the Raspberry Pi 4B environment, two testing methods are employed: video stream recording and real-time camera capture (Leng, 2022).

##### 4.6.2.1 Video input test

**Figure 13:**
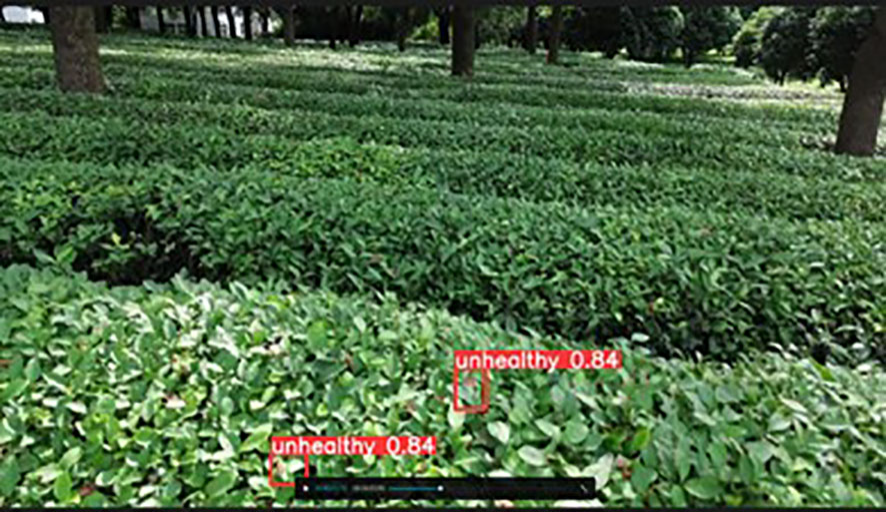
Detection results from the video.

The detection video was recorded when the image was collected on the afternoon of July 6, 2024, and was cropped to a duration of 5 sec. The video is transferred to the Raspberry Pi, which will detect tea pests and diseases in the video stream and save the identified content to the specified folder. As shown in **Figure 13**, the Raspberry Pi successfully identified the tea leaf blight in the video.

##### 4.6.2.2 Camera acquisition test

**Figure 14:**
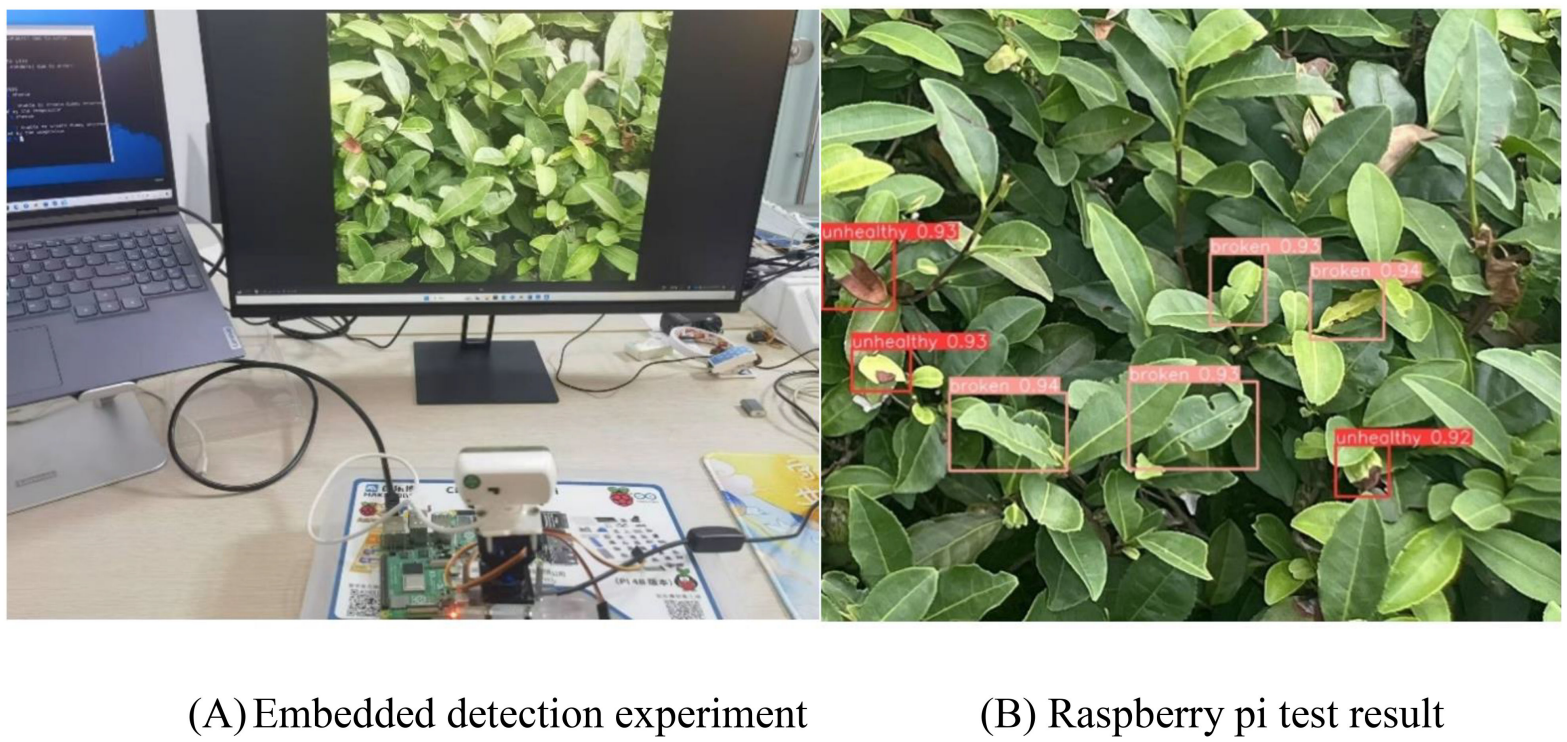
Detection of Embedded tea leaf disease symbols. **(A)** Embedded detection experiment, **(B)** Raspberry pi test result.

With Raspberry Pi as the core, the camera is used to monitor the pictures displayed on the PC. **Figure 14A** shows the experimental process of the embedded tea pest and disease detection system. **Figure 14B** is an enlarged screenshot of the detection result in **Figure 14A**. The experimental results show that Raspberry Pi can accurately identify tea pests and diseases on the display screen.

We successfully implemented and tested the WMC-RTDETR lightweight algorithm on cost-effective embedded devices. The tests on video streams and image data demonstrated that the Raspberry Pi accurately identified pests and diseases on tea leaves. This not only confirms the effectiveness of the algorithm in real-world applications, but also demonstrates the wide range of its applicability.

## 5 Conclusion

The WMC-RTDETR model proposed in this paper shows significant performance improvement in the tea disease detection task. The various components of the RT-DETR model were optimized and improved. The wavelet transform convolution module enhanced the model’s ability to capture multi-scale features by decomposing the input signal in the frequency domain. The multiscale multihead self-attention module overcomes the limitations of the traditional attention mechanism in small object detection; The context-guided spatial feature reconstruction feature pyramid network effectively alleviates the detection difficulties caused by complex background and target occlusion. The experimental results show that the improved model outperforms the original model in key metrics such as mAP50, FPS, and the number of parameters. In addition, the successful deployment of the WMC-RTDETR model on the embedded platform Raspberry Pi, a low-cost pest and disease detection system was realized. This deployment further validated the practicality and feasibility of the model and provided a practical solution for real-time disease monitoring in agricultural scenarios.

Although the experimental results prove the superiority of the model, there is still room for improvement. First, the robustness of the model under extreme environmental conditions, such as bright light or low-light environments, still needs to be further verified. Second, the generalization ability of the model can be limited due to the small size of the collected dataset and the fact that no experiments have been conducted on other datasets. Future research can further optimize model performance by introducing large-scale, diverse datasets and applying multiple data enhancement strategies. Furthermore, in terms of hardware deployment, although the model achieves accurate recognition on the Raspberry Pi, a low-cost embedded platform, there is still room for improvement in detection speed. The next step could explore the use of faster recognition strategies and higher performance hardware platforms in more complex real-time systems to further improve the overall performance and utility of the system.

## Data Availability

The original contributions presented in the study are included in the article/supplementary material. Further inquiries can be directed to the corresponding author.
